# Comparison of edible brown algae extracts for the inhibition of intestinal carbohydrate digestive enzymes involved in glucose release from the diet

**DOI:** 10.1017/jns.2020.56

**Published:** 2021-01-12

**Authors:** Maha Attjioui, Sinead Ryan, Aleksandra Konic Ristic, Thomas Higgins, Oscar Goñi, Eileen R. Gibney, Joanna Tierney, Shane O'Connell

**Affiliations:** 1 Shannon Applied Biotechnology Centre, Institute of Technology Tralee, Tralee, Ireland; 2 Marigot Ltd., Carrigaline, Ireland; 3 UCD Institute of Food and Health, University College Dublin, Dublin, Ireland

**Keywords:** Type II diabetes, α-Glucosidase, Seaweed, *Ascophyllum nodosum*, *Fucus vesiculosis*, *Undaria pinnatifida*, AFE, polyphenol-rich extract from *Aschophyllum nodosum* and *Fucus vesiculosus*, AFCE, combination of polyphenols from *Aschophyllum nodosum* and *Fucus vesiculosus* and chromium, HPAEC-PAD, high-performance anion exchange chromatography with pulsed amperometric detection, MANE, pure seaweed extract from *Ascophyllum nodosum*, PCA, principal component analysis, pNPG, 4-nitrophenyl-β-d-glucopyranoside, UPE, fucoidan-rich extract from *Undaria pinnatifida*

## Abstract

Type II diabetes is considered the most common metabolic disorder in the developed world and currently affects about one in ten globally. A therapeutic target for the management of type II diabetes is the inhibition of α- glucosidase, an essential enzyme located at the brush border of the small intestinal epithelium. The inhibition of α-glucosidase results in reduced digestion of carbohydrates and a decrease in postprandial blood glucose. Although pharmaceutical synthetic inhibitors are available, these are usually associated with significant gastrointestinal side effects. In the present study, the impact of inhibitors derived from edible brown algae is being investigated and compared for their effect on glycaemic control. Carbohydrate- and polyphenolic-enriched extracts derived from *Ascophyllum nodosum*, *Fucus vesiculosus* and *Undaria pinnatifida* were characterised and screened for their inhibitory effects on maltase and sucrase enzymes. Furthermore, enzyme kinetics and the mechanism of inhibition of maltase and sucrase were determined using linear and nonlinear regression methods. All tested extracts showed a dose-dependent inhibitory effect of α-glucosidase with IC_50_ values ranging from 0⋅26 to 0⋅47 mg/ml for maltase; however, the only extract that was able to inhibit sucrase activity was *A. nodosum*, with an IC_50_ value of 0⋅83 mg/ml. The present study demonstrates the mechanisms in which different brown seaweed extracts with varying composition and molecular weight distribution differentially inhibit α-glucosidase activities. The data highlight that all brown seaweed extracts are not equal in the inhibition of carbohydrate digestive enzymes involved in postprandial glycaemia.

## Introduction

The global prevalence of diabetes has doubled during the past 20 years and is currently affecting the health of millions of people^(1)^. Type II diabetes is the most common form of diabetes and is usually characterised by the presence of obesity and/or an abnormal increase of postprandial glycaemia, insulin resistance and relative insulin deficiency^(2,3)^. It is considered that the control of postprandial glycaemia is one of the strategies for the management of type II diabetes, through a reduction in the consumption of foods with high amounts of readily available carbohydrates^(4)^ or through an inhibition of the key enzymes involved in the digestion of carbohydrates^(5,6)^. In the human gastrointestinal tract, dietary carbohydrates are digested into glucose by six different enzymes: first by salivary and pancreatic α-amylase, also known as α-1,4-endoglucosidases, and then by the mucosal α-glucosidases maltase, sucrase, glucoamylase and isomaltase^(7,8)^. α-Glucosidases are located on the brush border membrane of the small intestine and form two complexes with different substrate specificities, maltase-glucoamylase and sucrase–isomaltase complexes^(9)^. Maltase is the major enzyme responsible for digestion and absorption of dietary starch. It hydrolyses the α-1,4-linkages of maltose residues to release a single glucose molecule, whereas sucrase hydrolyses the α-1,2-linkages of sucrose into glucose and fructose^(10)^. The glucose is then absorbed by the intestinal epithelial cells and then released into blood circulation to be used as an energy source for the human body ^(11)^. Therefore, the inhibition of maltase and sucrase enzymes in the gut can reduce postprandial glucose and help regulate glucose levels in the bloodstream after the ingestion of carbohydrate-rich meal^(12)^. In type II diabetes, oral antidiabetic drugs such as acarbose, miglitol and voglibose are known for inhibiting α-glucosidase activity^(13)^; however, some of these treatments come with side effects like abdominal distention and gas accumulation due to the undigested starch and sugar reaching the colon^(6)^. It has also been reported that long-term use of these drugs might result in more serious side effects, especially in patients with chronic renal failure^(14)^.

Thus, alternative products derived from natural sources, such as marine seaweed, have received significant interest in the last few years due to their promising health properties^(15,16)^. Research has been published on the bioactive properties of seaweeds and their extracts for numerous potential applications in human health and nutrition^(17–20)^. Seaweeds are rich in bioactive compounds in the form of polyphenols, carotenoids, vitamins, phycobilins, phycocyanins and polysaccharides, many of which had been shown to have an effect on glycaemic function^(21–23)^. For instance, polyphenols and, specifically, phlorotannins present in brown seaweeds exhibited strong α-amylase and α-glucosidase inhibition *in vitro*^(24)^. In addition, supplementation with polyphenolic-rich extracts has been reported to be effective for postprandial blood glucose control and significantly reduced fasting blood insulin levels in human subjects^(25)^. Increased insulin sensitivity in non-diabetic patients after the consumption of a seaweed polyphenol-rich extract has also been reported^(26)^. Fucoidan is an abundant bioactive sulphated polysaccharide in brown seaweed and has previously been shown to inhibit the starch-digesting enzymes, α-amylase and α-glucosidase^(27)^. In addition, fucosterol, a sterol found in brown seaweed, also reduced postprandial blood glucose levels and glycogen degradation when administered orally in epinephrine-induced diabetic rats^(28)^.

Despite this growing evidence, identification and selection of the most promising seaweeds and/or extracts is difficult, due to a deficit of comparable data, the use of different experimental models, extraction procedures, extract compositions and bioactive component physicochemical properties^(29,30)^. The availability of comparable compositional data would provide an initial understanding on the key biomolecules that are contributing to the efficacy in reducing postprandial hyperglycaemia. To our knowledge, most studies citing the effects of seaweed on the inhibition of α-glucosidases were conducted using artificial substrates such as 4-nitrophenyl-β-d-glucopyranoside (pNPG)^(31,32)^, and no study carried out to date has compared the effects of the composition and structure of seaweed extracts on their ability to inhibit maltase and sucrose activities. In the present study, we investigated the differential inhibitory effects of four brown seaweed extracts from different sources with varying composition and molecular weight distribution on maltase and sucrase activities. In addition, we determined the inhibition mechanisms and the respective inhibition constants for each of these extracts.

## Material and methods

### Seaweed extracts

Four seaweed extracts derived from different brown algae species were evaluated in the present study: (1) a polyphenol-rich extract from *Aschophyllum nodosum* and *Fucus vesiculosus* (AFE); (2) a combination of polyphenols from *A. nodosum* and *F. vesiculosus* and chromium (AFCE); (3) a pure seaweed extract from *A. nodosum* (MANE) and (4) a fucoidan-rich extract from *Undaria pinnatifida* (UPE). AFE, AFCE and UPE were purchased from online supplement websites, and MANE was provided as a gift by Marigot Ltd.

### Compositional analysis of the seaweed extracts

The four brown seaweed extracts were characterised in terms of their polyphenol, fucoidan, uronics, glucose and ash content which were identified as the major components in these extracts^(23)^. Total phenolics were determined using the Folin–Ciocalteu's phenol reagent, according to the method described by Zhang *et al.*^(33)^. Total uronic acids were determined using the Blumenkrantz and Asboe-Hansen Method^(34)^. Fucose, xylose, mannose, galactose, glucose and mannitol content were analysed using high-performance anion exchange chromatography with pulsed amperometric detection (HPAEC-PAD)^(35)^. Sulphate content was determined using the BaCl_2_–gelatin turbidimetry method^(35)^. Fucoidan content was calculated as the sum of fucose, sulphate and other monosaccharides such as xylose, mannose and galactose according to Rioux and Turgeon^(23)^.

### Molecular weight analysis of the seaweed extracts

The molecular weight (*M*_w_) distribution of carbohydrates of the four brown seaweed extracts was detected and measured using high-performance size exclusion chromatography with a refraction index detector (HPSEC-RID). The HPSEC Shimadzu system consisted of a system controller CBM-20A, a solvent delivery module LC-20AD, an online degasser DGU-20A5, an autosampler SIL-20ACHT, a refraction index detector (Varian Prostar 350 RID) and an LC workstation. HPSEC analysis was performed using PL aquagel-OH MIXED-H columns (8 μm, 300 × 7⋅5 mm; Agilent). The mobile phase (0⋅1 M NaAc/0⋅1 M Na_2_SO_4_ buffer, pH 7⋅8) was used as the isocratic elution at room temperature. The flow rate and injection volume were set to 1 ml/min and 40 μl, respectively. A molecular weight calibration curve was constructed with the retention time values of known dextran standards (Sigma-Aldrich, MO, USA). For the analysis of the extracts, the measurable range was divided into four segments (>100, 50–100, 10–50 and <10 kDa). An average *M*_w_ for each extract within each range was determined, and relative peak area values were calculated using the LCsolution software (Shimadzu, Ireland).

### α-Glucosidase preparation

About 300 mg of rat intestinal α-glucosidase (EC 3.2.1.48) acetone powder (Sigma-Aldrich) was dissolved in 10 ml of phosphate buffer (100 mm, pH 6⋅9). The solution was sonicated in an ice bath for 30 min and then centrifuged at 10 000 *g* for 20 min at 4°C. The resulting supernatant was used as a source of α-glucosidases for activity and inhibition assays outlined later. Protein concentrations of the enzyme mixtures were determined using the Bradford assay (Bio-Rad).

### Maltase and sucrase inhibitory activities

d-(+)-maltose monohydrate and sucrose were purchased from Sigma-Aldrich (St. Louis, MO, USA). Maltase and sucrase inhibitory activities were obtained according to the method of Akkarachiyasit *et al.*^(36)^ with slight modifications. Briefly, the maltase inhibitory activity of the brown seaweed extracts was determined by incubating 100 μl of the extracts at final concentrations (0⋅1, 0⋅2, 0⋅3, 0⋅4 and 0⋅5  mg/ml) or phosphate buffer (100 mm, pH 6⋅9) with 50 μl of the diluted enzyme (1 : 30). After pre-incubating the reaction mixture at 37°C, 50 μl of maltose at final concentrations (1⋅25, 2⋅5, 5, 7⋅5 and 10 mm) in phosphate buffer (100 mm, pH 6⋅9) was added to the mixture and incubated for 30 min at 37°C. For sucrase inhibition, 100 μl of seaweed extracts (0⋅6, 0⋅7, 0⋅8, 0⋅9 and 1  mg/ml) or phosphate buffer (100 mm, pH 6⋅9) were incubated with 50 μl of the diluted enzyme (1 : 5). After pre-incubating the reaction mixture at 37°C, 50 μl of sucrose at final concentrations (15, 17⋅5, 20, 22⋅5 and 25 mm) in phosphate buffer (100 mm, pH 6⋅9) was added to the mixture and incubated for 60 min at 37°C. The reaction mixtures were then heated at 100°C for 10 min to stop the reaction and centrifuged at 1000 *g* at 4°C to precipitate the enzyme. Acarbose was used as a positive control for maltase inhibition at final concentrations of 0⋅0001–0⋅001  mg/ml.

After the determination of the released glucose, the inhibitory activity was calculated from the formula as follows:

Inhibition (%) = (*C* − *T*)/*C* × 100, where *C* is the enzyme activity without the inhibitor and *T* is the enzyme activity with the inhibitor.

### HPAEC-PAD and glucose oxidase for glucose quantification

Quantitative analysis of glucose after the enzymatic reaction was carried out by using two different detection methods: direct HPAEC–PAD or indirect enzymatic measurement with the glucose oxidase method using d-Glucose Assay Kit (Megazyme, Bray, Ireland). For the HPAEC–PAD analysis, samples were diluted with deionised water to fall within the calibration range, filtered through a 0⋅2 μM PTFE filter and maintained at 4°C before analysis. Glucose was separated using a CarboPac PA-1 anion-exchange resin column (4⋅6 × 250 mm) connected to a CarboPac PA-1 guard column (4⋅6 × 50 mm) (Thermo Scientific Dionex, Ireland) at 18°C in an isocratic gradient (18 mm NaOH). The chromatography system consisted of an Agilent 1260 Infinity Quaternary LC system (Agilent, Ireland) and a Decade II electrochemical detector (Antec Leyden, Netherlands). The amperometry detector cell contained a gold electrode and a HyREF reference electrode. Glucose was identified by a comparison of the retention time to that of the commercial standard (Sigma-Aldrich, MO, USA) and quantified by the integration of peak area with the ChemStation software (Agilent, Ireland).

### Determination of IC_50_ values

The IC_50_ value was defined as the concentration of each inhibitor to inhibit 50  per cent of the rat maltase and sucrase activities from rat intestinal α-glucosidase. It was determined by linear regression of the log-transformed values of inhibitor concentration (log [*I*]) *v.* the relative activity (*v*/*V*_max_) at 10 mm maltose, and 25 mm sucrose using GraphPad Prism software methods (GraphPad Prism ver. 6, GraphPad Software, La Jolla, CA, USA).

### Determination of inhibition constants and mechanism of inhibition

Enzyme kinetic assays for maltase activity were performed according to the reaction conditions described earlier with varying concentrations of the substrate maltose (1⋅25, 2⋅5, 5, 7⋅5 and 10 mm). Inhibition/dissociation constants *K* (*K_i_* or *K_i_*′) were determined using Dixon and Cornish-Bowden plots^(37)^, fitting obtained linear data through Microsoft Office Excel v.2013. *K_i_* constants were determined for inhibitors that showed competitive and mixed inhibition using Dixon plots, and the *K_i_*′ constants were determined by a Cornish-Bowden plot for inhibitors that showed uncompetitive inhibition, non-competitive inhibition and mixed inhibition. The ratio *K_i_*′/*K_i_* was used to determine the mechanism of inhibition, because its value established the degree to which the binding of inhibitors changes the affinity of the enzyme for the substrate^(38,39)^.

### Statistical analysis

All experiments were conducted in triplicate, and results were expressed as mean values ± standard error (se). Statistically significant differences were analysed with the one-way analysis of variance (ANOVA) by Tukey's HSD test at *P* ≤ 0⋅05 using Sigma Plot 12.0 (Systat Software, Inc., San Jose, CA, USA) and Statgraphics Centurion XVI (Statgraphics Technologies, Inc., The Plains, VA, USA) software. The application of this parametric test was performed after checking the data normality (Shapiro–Wilk's test) and equal variance assumptions. Principal component analysis (PCA) was performed to establish if a correlation existed between the composition, molecular weight distribution and the maltase activity inhibition (expressed as IC_50_). The PCA was assessed using XLSTAT software package version 2014.5.03 (www.xlstat.com; Addinsoft, New York, NY, USA) through the correlation matrix Pearson *n*−1. The correlation biplot was determined on the basis of the first and second principal components (PCs).

## Results

### Composition and molecular weight analysis of seaweed extracts

Polyphenols, fucoidan, glucose, uronics and minerals were identified and analysed as the key components of the brown seaweed extracts (Table 1). AFE had the highest uronics content, about 3-fold higher than AFCE and UPE, and 11-fold higher than MANE, the second highest fucoidan content after UPE, the second highest polyphenol content after AFCE and double the mannitol content of the rest of the extracts. While AFCE contained mainly polyphenols, which was 2-fold higher than AFE, it also had the highest glucose content and the lowest fucoidan and mineral contents. MANE was high in minerals and fucoidan, having 2-fold higher polyphenol content than UPE and the lowest uronic content. Finally, UPE had the highest fucoidan content, approximately 2-fold greater than MANE and 3-fold greater than AFE. UPE had the second highest ash content with similar levels to AFE. UPE also contained a similar uronic content to AFCE and had the lowest polyphenol and glucose content of all extracts (Table 1).

**Table 1.** Compositional analysis of four seaweed extracts

	Fucoidan (w/w%)	se	Mannitol (w/w%)	se	Glucose (w/w%)	se	Uronics (w/w%)	se	Ash (w/w%)	se	Polyphenols (w/w %)	se	Others (w/w%)
AFE	24⋅43^a^	0⋅02	0⋅99^a^	0⋅01	0⋅45^a^	0⋅01	28⋅46^a^	0⋅53	11⋅42^ac^	1⋅67	12⋅30^a^	0⋅27	21⋅95
AFCE	14⋅86^b^	0⋅09	0⋅47^bd^	0⋅13	10⋅41^b^	0⋅02	9⋅36^b^	0⋅46	2⋅26^a^	1⋅04	23⋅54^b^	0⋅17	39⋅1
MANE	19⋅34^c^	0⋅08	0⋅57^cb^	0⋅01	0⋅27^ac^	0⋅01	2⋅48^c^	0⋅06	58⋅80^b^	1⋅77	6⋅57^c^	0⋅19	11⋅97
UPE	43⋅60^d^	0⋅13	0⋅43^cd^	0⋅02	0⋅24^c^	0⋅06	8⋅33^b^	0⋅09	15⋅48^c^	4⋅49	3⋅15^d^	0⋅08	28⋅77

se, standard error; AFE, polyphenol-rich extract from *Aschophyllum nodosum* and *Fucus vesiculosus*; AFCE, combination of polyphenols from *Aschophyllum nodosum* and *Fucus vesiculosus* and chromium; MANE, pure seaweed extract from *Ascophyllum nodosum*; UPE, fucoidan-rich extract from *Undaria pinnatifida*.

Values with different superscript letters within the same column are significantly different using Tukey's HSD test at *P* ≤ 0⋅05 (*N* 3).

The *M*_w_ distribution values of the brown seaweed extracts outlined in Table 2 showed that AFE and AFCE were mainly composed of high *M*_w_ biomolecules (>100 kDa) with an average *M*_w_ of ~800 kDa, representing between 54 and 70  per cent of the total biomolecules detected, respectively. However, MANE and UPE were characterised as containing a significant proportion of lower *M*_w_ biomolecules (<50 kDa) with average molecular weights of 13⋅66 and 20⋅61 kDa, respectively. Biomolecules detected in the 1–10 kDa range for MANE were approximately 3-fold more abundant compared with UPE (Table 2).

**Table 2.** Molecular weight distribution analysis of four seaweed extracts expressed as the average value of the main peak areas or the relative peak area in four *M*_w_ range values

	Average *M*_w_							
	>100 kDa	se	50–100 kDa	se	10–50 kDa	se	1–10 kDa	se
AFE	793⋅33^b^	4⋅16	98⋅25^a^	3⋅293	50⋅42^a^	0⋅01	5⋅60^a^	0⋅08
AFCE	799⋅30^b^	20⋅71	101⋅39^a^	0⋅193	50⋅18^a^	0⋅04	3⋅96^c^	0⋅05
MANE	567⋅84^a^	1⋅42	50⋅70^b^	0⋅188	13⋅66^b^	0⋅90	7⋅58^b^	0⋅03
UPE	564⋅83^a^	0⋅85	50⋅61^b^	0⋅076	20⋅61^c^	0⋅16	7⋅56^b^	0⋅02
	Area (%)^1^							
	>100 kDa	se	50–100 kDa	se	10–50 kDa	se	1–10 kDa	se
AFE	70⋅75^a^	1⋅94	10⋅65^a^	0⋅78	11⋅09^a^	0⋅73	8⋅43^a^	0⋅75
AFCE	54⋅01^d^	2⋅65	8⋅87^c^	0⋅24	12⋅17^a^	1⋅32	24⋅96^b^	1⋅1
MANE	20⋅86^b^	2⋅68	11⋅84^a^	0⋅27	38⋅17^b^	0⋅75	29⋅14^b^	2⋅18
UPE	32⋅40^c^	0⋅55	13⋅36^b^	0⋅02	42⋅92^c^	0⋅64	11⋅32^a^	0⋅21

se, standard error; AFE, polyphenol-rich extract from *Aschophyllum nodosum* and *Fucus vesiculosus*; AFCE, combination of polyphenols from *Aschophyllum nodosum* and *Fucus vesiculosus* and chromium; MANE, pure seaweed extract from *Ascophyllum nodosum*; UPE, fucoidan-rich extract from *Undaria pinnatifida.*

Values with different superscript letters within the same column are significantly different using Tukey's HSD test at *P* ≤ 0⋅05. Peak areas corresponding to specific *M*_w_ values (*N* 3).

1 Peak areas were calculated for the specific *M*_w_ ranges described earlier.

### Maltase, sucrase inhibitory activity and IC_50_ of seaweed extracts

**Fig. 1.**
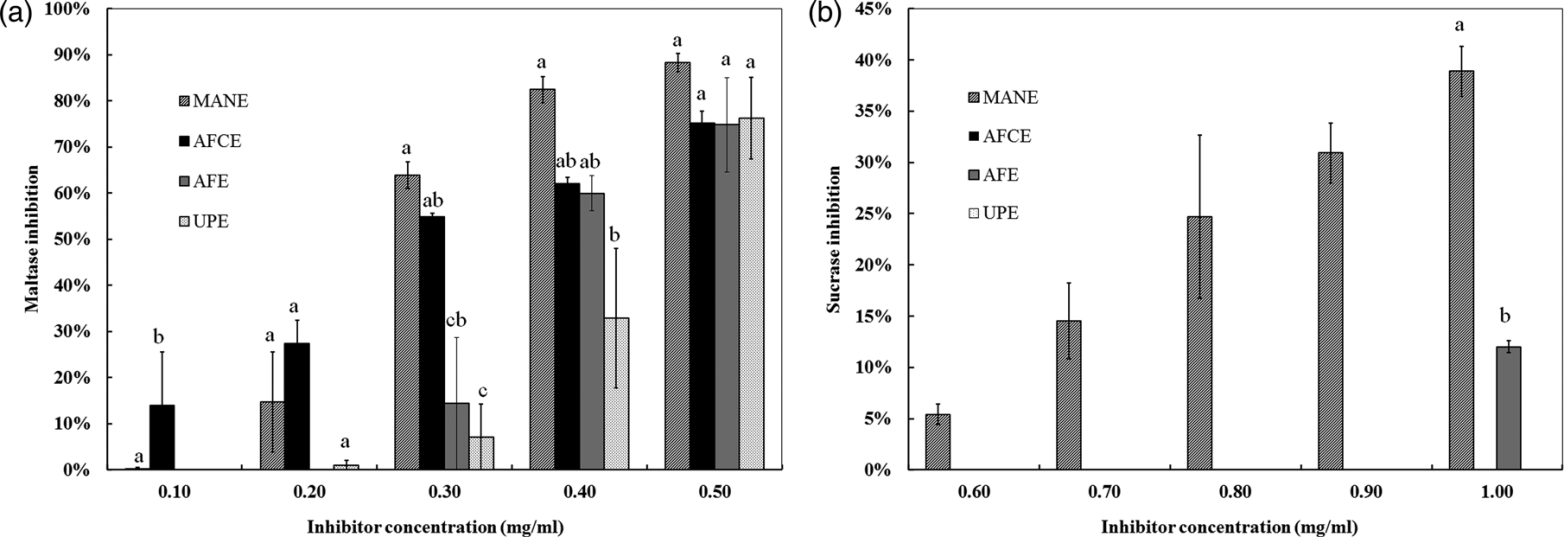
Inhibitory activities of different brown seaweed extracts on the activities of maltase and sucrase. (a) Maltase inhibition using 10 mm maltose as a substrate. (b) Sucrase inhibition using 25 mm sucrose as a substrate. Released glucose was determined using high-performance anion exchange chromatography with pulsed amperometric detection (HPAEC-PAD). Data represent the average of *n* 3 and were subjected to one-way analysis of variance and Tukey's HSD test for evaluating the differences among means at *P* ≤ 0⋅05. AFE, polyphenol-rich extract from *Aschophyllum nodosum and Fucus vesiculosus*; AFCE, combination of polyphenols from *Aschophyllum nodosum* and *Fucus vesiculosus* and chromium; MANE, pure seaweed extract from *Ascophyllum nodosum*; UPE, fucoidan-rich extract from *Undaria pinnatifida*.

The inhibitory effect of the four brown seaweed extracts was assessed separately *in vitro* on maltase and sucrase activity at various concentrations. The inhibition rates for maltase ranged from 75 to 88  per cent at the highest concentration of 0⋅5 mg/ml, while only 36  per cent inhibition was observed for sucrase at 1 mg/ml, with a linear dose response in the whole range of the tested concentrations (Fig. 1). Significant differences between MANE and UPE were observed at 0⋅4 mg/ml for maltase inhibition, and between MANE and UPE, and between MANE and AFE at 0⋅3 mg/ml (Fig. 1(A)). The IC_50_ value for MANE was 0⋅26 mg/ml, which was significantly lower than UPE 0⋅47 mg/ml. No significant difference was observed between AFE, AFCE and MANE (IC_50_ between 0⋅26 and 0⋅33 mg/ml) (Table 3), while MANE was the strongest inhibitor of sucrase activity with 36  per cent inhibition at 1 mg/ml and IC_50_ of 0⋅83 mg/ml. The other extracts and acarbose were not found to inhibit sucrase (Table 3).

**Table 3.** IC_50_ values for the inhibition of maltase (10 mm) and sucrase (25 mm) enzymes by the different seaweed extracts as determined by HPAEC-PAD and the enzymatic method

Inhibitor	IC_50_ (mg/ml)							
	HPAEC-PAD				Glucose oxidase			
	Maltase	se	Sucrase	se	Maltase	se	Sucrase	se
AFE	0⋅33^ab^	0⋅02	ND	–	0⋅32^a^	0⋅01	ND	–
AFCE	0⋅28^ab^	0⋅05	ND	–	0⋅20^b^	0⋅01	ND	–
MANE	0⋅26^a^	0⋅01	0⋅83	0⋅12	0⋅25^ab^	0⋅01	ND	–
UPE	0⋅47^b^	0⋅03	ND	–	0⋅50^c^	0⋅02	ND	–
Acarbose^1^	0⋅15 × 10^−3^	0⋅02 × 10^−3^	ND	–	–	–	–	–

se, standard error; ND, not detected; AFE, polyphenol-rich extract from *Aschophyllum nodosum* and *Fucus vesiculosus*; AFCE, combination of polyphenols from *Aschophyllum nodosum* and *Fucus vesiculosus* and chromium; MANE, pure seaweed extract from *Ascophyllum nodosum*; UPE, fucoidan-rich extract from *Undaria pinnatifida.*

Values with different superscript letters are significantly different using Tukey's HSD test at *P* ≤ 0⋅05

1 Positive control for maltase inhibition (*N* 6).

### Mechanism of maltase inhibition by seaweed extracts

**Fig. 2.**
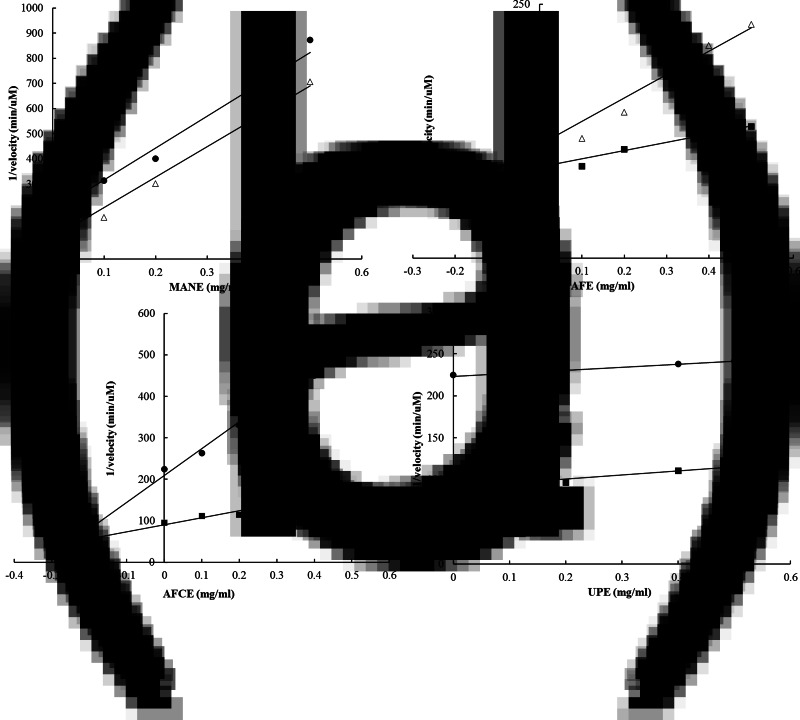
Dixon plots for the determination of the type of inhibition of maltase by different brown seaweed extracts. (a) MANE, a pure seaweed extract from *Ascophyllum nodosum.* (b) AFE, a polyphenol-rich extract from *Aschophyllum nodosum* and *Fucus vesiculosus.* (c) AFCE, a combination of polyphenols from *Aschophyllum nodosum* and *Fucus vesiculosus* and chromium. (d) UPE, a fucoidan-rich extract from *Undaria pinnatifida.* The concentrations of maltose used were 1⋅25 mm (•), 2⋅5 mm (Δ) and 5 mm (■). Data represent the average of *n* 3.

The inhibition mechanism and respective kinetic constants on maltase activity were determined for each brown seaweed extract. The glucose produced from the enzymatic reaction was detected using the HPAEC–PAD analysis. The *K_i_* constants were measured using Dixon plots, and the *K_i_*′ constants were determined by using a Cornish-Bowden plot (Table 4). All obtained linear equations had a coefficient of determination (*r*^2^) higher than 0⋅85 for substrate concentrations ranging from 1⋅25 to 5 mm. According to the calculated ratio *K_i_*′/*K_i_*, the four brown seaweed extracts were found to have different mechanisms of action in inhibiting maltase activity. AFE behaved as a competitive inhibitor (*K_i_*'/*K_i_* = infinite) (Fig. 2(b)), MANE and UPE were characterised as uncompetitive inhibitors (*K_i_*'/*K_i_* = 0) (Figs. 2(a) and 2(d)) and AFCE worked as a mixed inhibitor (*K_i_*'/*K_i_* > 1) (Fig. 2(c)). Acarbose was analysed as a positive control and was confirmed as a competitive inhibitor (see Supplementary material).

**Table 4.** Evaluation of mechanism of maltase inhibition by four seaweed extracts

	AFE	AFCE	MANE	UPE	Acarbose*
*K_i_* (mg/ml)	0⋅15	0⋅22	Infinite	Infinite	0⋅07 × 10^−3^
*K_i_*′ (mg/ml)	Infinite	0⋅25	0⋅12	0⋅81	Infinite
*K_i_*′/*K_i_*	Infinite	>1	0	0	Infinite
Model	Competitive	Mixed	Uncompetitive	Uncompetitive	Competitive

AFE, polyphenol-rich extract from *Aschophyllum nodosum* and *Fucus vesiculosus*; AFCE, combination of polyphenols from *Aschophyllum nodosum* and *Fucus vesiculosus* and chromium; MANE, pure seaweed extract from *Ascophyllum nodosum*; UPE, fucoidan-rich extract from *Undaria pinnatifida.*

* Positive control for maltase inhibition.

### Principal component analysis

**Fig. 3.**
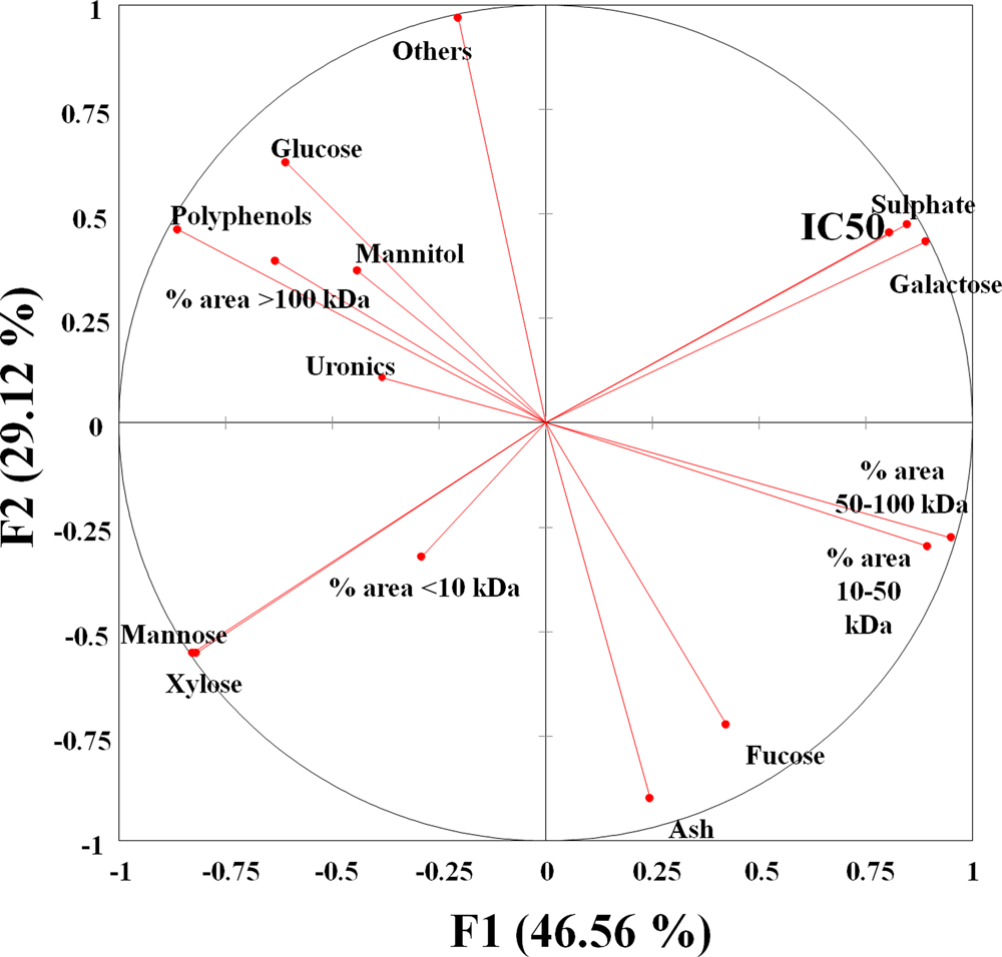
Principal component analysis (PCA) correlation biplot based on the first two principal components (PC1 and PC2) generated from the analysed compositional and *M*_w_ parameters of the soluble bioactive components of the brown seaweed extracts and the maltase activity inhibition (expressed as IC_50_).

PCA was performed to establish if a relationship existed between extract composition, physicochemical parameters and the observed maltase inhibition activity. The first two PCs from the correlation biplot explained 75⋅68  per cent of the total variance, with PC1 and PC2 accounting for 46⋅56 and 29⋅12  per cent, respectively (Fig. 3). A strong negative correlation between mannose and xylose content released from the fucoidan polymer/oligomer and maltase IC_50_ was observed in the statistical Pearson correlation test, with correlation coefficient values of −0⋅850 and −0⋅877, respectively (Table 5). Opposite correlation coefficient values of similar magnitude were found between two relative peak area parameters (1–10 and 50–100 kDa) and IC_50_, which indicates that there is a relationship between *M*_w_ and maltase inhibition. A relationship between higher maltase IC_50_ values and higher content of galactose and sulphate is indicated, with correlation coefficient values of 0⋅965 and 0⋅989, respectively (Table 5), suggesting that these components do not have a role in inhibiting maltase.

**Table 5.** Correlation matrix between seaweed extract composition, *M*_w_ parameters and IC_50_ for maltase inhibition

Variables	Mannitol	Glucose	Uronics	Ash	Polyphenols	Others	IC_50_	Fucose	Galactose	Mannose	Xylose	Sulphate	% Area <10 kDa	% Area 10–50 kDa	% Area 50–100 kDa	% Area >100 kDa
Mannitol	1	−0⋅359	0⋅881	−0⋅080	0⋅047	−0⋅375	−0⋅212	0⋅525	−0⋅446	0⋅695	0⋅656	−0⋅356	−0⋅495	−0⋅561	−0⋅203	0⋅686
Glucose	−0⋅359	1	−0⋅148	−0⋅530	0⋅914	0⋅797	−0⋅393	−0⋅976	−0⋅337	0⋅076	0⋅108	−0⋅335	0⋅419	−0⋅567	−0⋅821	0⋅301
Uronics	0⋅881	−0⋅148	1	−0⋅527	0⋅195	0⋅058	0⋅088	0⋅262	−0⋅175	0⋅402	0⋅357	−0⋅057	−0⋅752	−0⋅680	−0⋅263	0⋅898
Ash	−0⋅080	−0⋅530	−0⋅527	1	−0⋅553	−0⋅870	−0⋅350	0⋅551	−0⋅219	0⋅236	0⋅252	−0⋅306	0⋅546	0⋅606	0⋅428	−0⋅748
Polyphenols	0⋅047	0⋅914	0⋅195	−0⋅553	1	0⋅654	−0⋅561	−0⋅810	−0⋅594	0⋅418	0⋅437	−0⋅558	0⋅284	−0⋅842	−0⋅977	0⋅592
Others	−0⋅375	0⋅797	0⋅058	−0⋅870	0⋅654	1	0⋅224	−0⋅861	0⋅219	−0⋅388	−0⋅378	0⋅255	−0⋅126	−0⋅424	−0⋅482	0⋅415
IC_50_	−0⋅212	−0⋅393	0⋅088	−0⋅350	−0⋅561	0⋅224	1	0⋅220	0⋅965	−0⋅850	−0⋅877	0⋅989	−0⋅720	0⋅462	0⋅695	−0⋅081
Fucose	0⋅525	−0⋅976	0⋅262	0⋅551	−0⋅810	−0⋅861	0⋅220	1	0⋅136	0⋅140	0⋅106	0⋅144	−0⋅386	0⋅410	0⋅679	−0⋅182
Galactose	−0⋅446	−0⋅337	−0⋅175	−0⋅219	−0⋅594	0⋅219	0⋅965	0⋅136	1	−0⋅948	−0⋅963	0⋅993	−0⋅514	0⋅628	0⋅747	−0⋅310
Mannose	0⋅695	0⋅076	0⋅402	0⋅236	0⋅418	−0⋅388	−0⋅850	0⋅140	−0⋅948	1	0⋅999	−0⋅918	0⋅267	−0⋅624	−0⋅603	0⋅413
Xylose	0⋅656	0⋅108	0⋅357	0⋅252	0⋅437	−0⋅378	−0⋅877	0⋅106	−0⋅963	0⋅999	1	−0⋅938	0⋅317	−0⋅616	−0⋅620	0⋅384
Sulphate	−0⋅356	−0⋅335	−0⋅057	−0⋅306	−0⋅558	0⋅255	0⋅989	0⋅144	0⋅993	−0⋅918	−0⋅938	1	−0⋅609	0⋅541	0⋅708	−0⋅196
% Area <10 kDa	−0⋅495	0⋅419	−0⋅752	0⋅546	0⋅284	−0⋅126	−0⋅720	−0⋅386	−0⋅514	0⋅267	0⋅317	−0⋅609	1	0⋅135	−0⋅316	−0⋅544
% Area 10–50 kDa	−0⋅561	−0⋅567	−0⋅680	0⋅606	−0⋅842	−0⋅424	0⋅462	0⋅410	0⋅628	−0⋅624	−0⋅616	0⋅541	0⋅135	1	0⋅886	−0⋅905
% Area 50–100 kDa	−0⋅203	−0⋅821	−0⋅263	0⋅428	−0⋅977	−0⋅482	0⋅695	0⋅679	0⋅747	−0⋅603	−0⋅620	0⋅708	−0⋅316	0⋅886	1	−0⋅615
% Area >100 kDa	0⋅686	0⋅301	0⋅898	−0⋅748	0⋅592	0⋅415	−0⋅081	−0⋅182	−0⋅310	0⋅413	0⋅384	−0⋅196	−0⋅544	−0⋅905	−0⋅615	1

## Discussion

It is widely accepted that the control of postprandial glycaemia is an effective strategy for the management of type II diabetes^(5,6)^. The inhibition of mucosal intestinal enzymes maltase and sucrase, two key enzymes involved in the breakdown of carbohydrates and intestinal absorption of glucose, can slow down the release of glucose into the blood and significantly decrease postprandial glycaemia^(13)^. Seaweed extracts have been previously reported as potential inhibitors of α-glucosidases and thereby as an alternative to synthetic drugs^(19,22)^. In the present study, we investigated the inhibitory effects of four well-characterised brown seaweed extracts and revealed their mechanism of inhibition on maltase and sucrase enzymes. In addition, we utilised regression analysis to investigate the relationship between their chemical composition, *M*_w_ distribution and their maltase inhibition (IC_50_ value).

The compositional analysis revealed significant differences in the biomolecule contents of the extracts and their molecular weight distributions. The differences observed were expected since the composition and structure of different seaweeds are known to vary depending on the species, location, season of harvest and extraction method^(29,40,41)^. More importantly, many researchers have reported the important role that these parameters play in the determination of the bioactivity of seaweed^(42)^. For example, Lordan *et al.* reported that the extraction solvent ratio affected the composition of the phenolic compounds and consequently the α-glucosidase inhibitory effects of the extracts^(43)^. The four brown seaweed extracts within the present study were tested for their abilities to inhibit maltase and sucrase enzymatic activities. Results obtained from glucose detected both through HPAEC–PAD and the glucose oxidase method showed that all extracts significantly inhibited maltase activity in a concentration-dependent manner, but only the *A. nodosum* extract (MANE) was able to inhibit both maltase and sucrase activities. The ability of MANE to inhibit both maltose and sucrose hydrolysis suggests that it may be more robust and effective in the reduction of postprandial hyperglycaemia. The most popular method used to date for the detection of glucose in seaweed extract α-glucosidase inhibition studies is glucose oxidase or hexokinase assays. However, we found that the levels of glucose detected in the enzyme reaction mixture using the glucose oxidase method were much lower compared with HPAEC-PAD (data not shown). Although IC_50_ values for maltase inhibition were close for both methods, the chromatographic analytical method was more sensitive in detecting glucose in the sucrase inhibition assay. It has been reported previously that polyphenols can have a scavenger effect on the oxidative intermediates generated during the glucose oxidase–peroxidase reaction, which can lead to possible misleading inhibition data and/or an underestimation of glucose results^(44)^. Therefore, HPAEC–PAD seems to be a more accurate analytical method for the measurement of glucose in α-glucosidase inhibition studies, especially in those natural extracts with a significant amount of polyphenolic and/or antioxidant components.

The inhibitory effect of edible seaweeds on α-glucosidase has been previously reported using the artificial substrate pNPG^(31,32)^, which does not have the ability to discriminate between maltase and sucrase inhibition activities. The amount of sucrose and maltose can vary in an individual's diet, and this variation can impact on the effectiveness of α-glucosidase inhibition on postprandial hyperglycaemia. There is a limited number of recent studies reporting the effects of seaweed/seaweed extracts on maltase or sucrase inhibition. Hwang *et al.* showed that extracts from the brown seaweed *Sargassum hemiphyllum* were more efficient at inhibiting maltase (IC_50_: 0⋅09–2⋅88 mg/ml) than sucrase (IC_50_: 1⋅89–3⋅47 mg/ml)^(45)^. Other studies have reported that bromophenols extracted from different red seaweed species such as *Polyopes lancifolia*, *Grateloupia* and *Symphyocladia latiuscula* inhibited rat-intestinal maltase (IC_50_: 1⋅2–5 mm) and sucrase (IC_50_: 1⋅0–4⋅2 mm)^(46–48)^. The measured IC_50_ values for maltase for the four brown seaweed extracts tested in the present study were in the low range (high potency) of the values mentioned previously. The MANE extract displays significant maltase and sucrase inhibition and is one of the most effective reported to date. The observed variation in IC_50_ reported in the literature could be linked to the use of specific experimental conditions, seaweed species, extraction procedures, extract composition and bioactive component physicochemical properties^(45,49,50)^.

The *K*_m_ value for maltase activity obtained using the Lineweaver–Burk plot was 5⋅8 mm and was found within the reported range, confirming the suitability of the *in vitro* experimental system^(23,51,52)^. To better understand how the four brown seaweed extracts inhibited maltase activity, the ratio *K_i_*′/*K_i_* was used to determine the mechanism of inhibition. When *K_i_*′/*K_i_* = 1, the inhibitor does not alter the substrate binding to the enzyme, and the model is identical to a non-competitive inhibition. However, when the *K_i_*′/*K_i_* is infinite, the binding of the inhibitor prevents the binding of the substrate and the model becomes identical to competitive inhibition. When *K_i_*′/*K_i_* is very small or equal to zero, the binding of the inhibitor enhances substrate binding to the enzyme, and the model becomes nearly identical to an uncompetitive model. Finally, when *K_i_*′/*K_i_* > 1, the model becomes a mixed inhibition^(38,39)^. The competitive inhibition mechanism from AFE would suggest that the substrate and the inhibitor cannot bind to the enzyme at the same time, competing simultaneously for access to the enzyme active site. This mechanism could have important implications in an *in vivo* scenario in the small intestine, as maltose is continuously released from the hydrolysis of dietary starch, and the effect of a competitive inhibitor can be diluted out by increasing amounts of the substrate. However, in the uncompetitive inhibition model (observed for MANE and UPE), the inhibitor binds to the substrate–enzyme complex and the inhibition is independent of the substrate concentration^(51)^. This reduces the influence of maltose accumulation on the efficacy of these inhibitors as they do not bind to the active site of the enzyme. Therefore, uncompetitive inhibitors have been reported to be more effective in controlling metabolic pathways *in vivo* than competitive inhibitors, making this mode of action more desirable^(52)^.

Fucoidans have been characterised as efficient α-amylase and α-glucosidase inhibitors, and their bioactivity has been reported to vary according to the seaweed species, harvest time, location, composition and physicochemical parameters of these carbohydrates such as *M*_w_, the number of sulphate groups and linkage position^(53–56)^. However, very little is known about the effects of the monosaccharide building blocks within the fucoidan polymer structure on α-glucosidase inhibition. In the present study, we found that specific compositional parameters of fucoidan molecules were correlated to maltase inhibitory activity. A higher content of xylose and mannose present in the fucoidan structure was correlated to lower IC_50_ values. However, the opposite was observed for sulphate and galactose, which increased IC_50_, and an extract with high amounts of these components would not be a good inhibitor. Our results would also suggest that there is a link between low *M*_w_ biomolecules in the tested brown seaweed extracts and high maltase inhibitory activity. This is consistent with the enhanced *in vitro* effects of low *M*_w_ fucoidans from brown seaweeds on inflammatory processes^(57)^.

In summary, it is evident from the data generated in the present study that there are significant compositional and structural differences between the four brown seaweed extracts studied. These differences appear to have a significant role on the profiles of enzyme activities inhibited and the type of inhibition mechanism. The pure seaweed extract from *A. nodosum* (MANE) was the best overall performer in terms of its potential to reduce the glucose released by intestinal enzymes for both starch- and carbohydrate-rich diets. The reported data also provide a better understanding of what is responsible for the inhibitory effects of seaweed extracts on these enzymes. This information should be of value in the evaluation and assessment of the potential use of different seaweed extracts in the control of postprandial hyperglycaemia.

## Supplementary material

For supplementary material accompanying this paper visit https://doi.org/10.1017/jns.2020.56. click here to view supplementary material
