# Supplementary material for: Comparison of edible brown algae extracts for the inhibition of intestinal carbohydrate digestive enzymes involved in glucose release from the diet
Source: J Nutr Sci. 2021 Jan 12;10:e5. doi: 10.1017/jns.2020.56 (PMC8057513; doi:10.1017/jns.2020.56)
Supplement: Supplementary file 1 [file S2048679020000567sup001.docx]

Supplementary

**.**

**Fucose**

**Mannitol**

**Xylose**

**Mannose**

**Galactose**

**Glucose**

**Normalized nC response**

**Elution time in ml**

**Figure S1: Chromatogram of the different elution times for the monosaccharides analyzed with HPAEC-PAD**

**Table S1: Inhibition percentages of maltase activities by different seaweed extracts**

| Inhibitor concentration (mg/ml) | % Inhibition | | | | | | | | |
| --- | --- | --- | --- | --- | --- | --- | --- | --- | --- |
|  | MANE | SE | UPE | SE | AFE | SE | AFCE | SE |  |
| 0.1 | +1 | 0.01 | -6 | 0.02 | -36 | 0.04 | +14 | 0.11 |  |
| 0.2 | +15 | 0.11 | +1 | 0.01 | -31 | 0.02 | +27 | 0.05 |  |
| 0.3 | +64 | 0.02 | +7 | 0.07 | +14 | 0.14 | +55 | 0.01 |  |
| 0.4 | +82 | 0.02 | +33 | 0.15 | +60 | 0.04 | +62 | 0.14 |  |
| 0.5 | +88 | 0.01 | +77 | 0.08 | +75 | 0.1 | +75 | 0.03 |  |

**
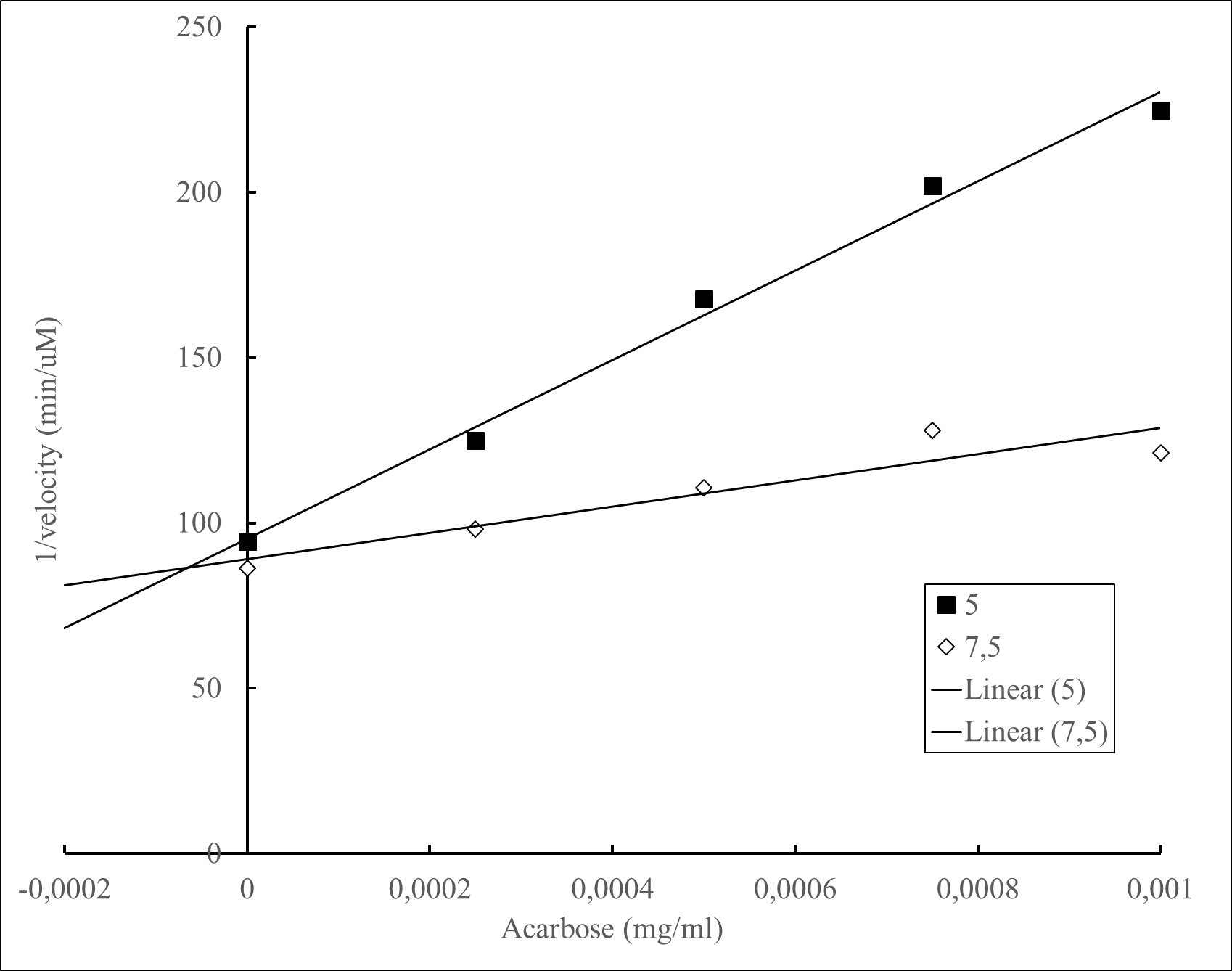
**

**Figure S2. Dixon plots for determination of the type of inhibition of maltase by Acarbose. The concentrations of Maltose used were 5 mM (▪), and 7.5 mM (◊). Data represent the average of n=3.**

**
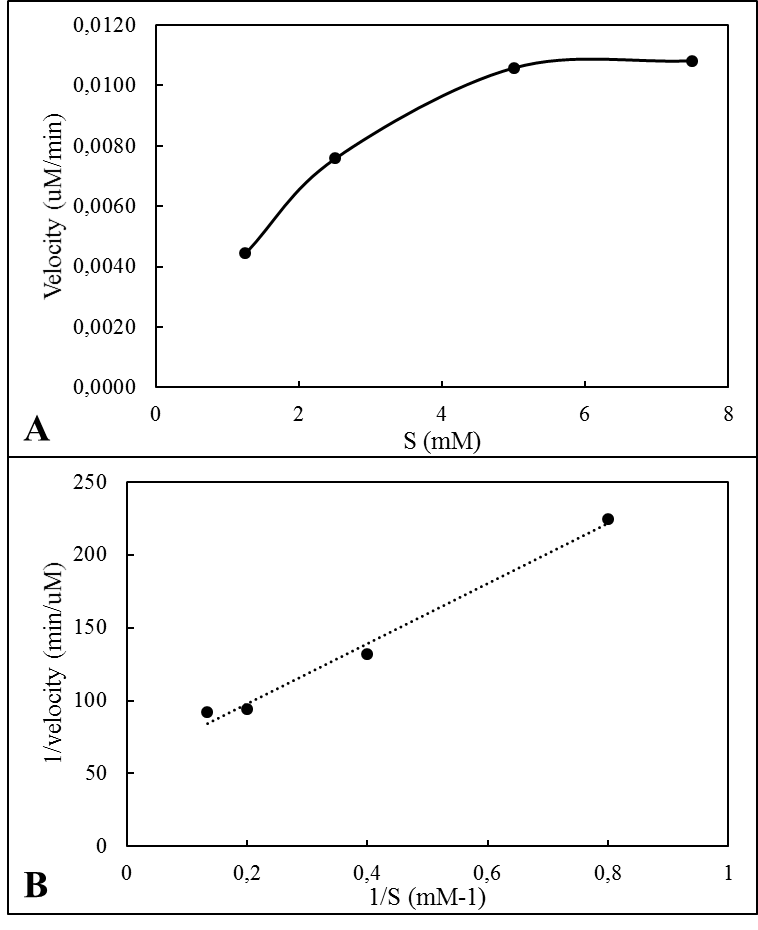
**

**Figure S3: A) plot of velocity vs Substrate concentration for maltase inhibition B) Lineweaver-Burk plot for maltase inhibition.**
